# Investigating the upsurge of malaria prevalence in Zambia between 2010 and 2015: a decomposition of determinants

**DOI:** 10.1186/s12936-019-2698-x

**Published:** 2019-03-07

**Authors:** Mukumbuta Nawa, Peter Hangoma, Andrew P. Morse, Charles Michelo

**Affiliations:** 1 School of Public Health, University of Zambia, Ridgeway Campus, Lusaka, Zambia; 2 Department of Geography and Planning, University of Liverpool, Liverpool, UK

**Keywords:** Malaria prevalence, Upsurge, Insecticide treated nets, Indoor residual spraying, Standard housing

## Abstract

**Background:**

Malaria is among the top causes of mortality and morbidity in Zambia. Efforts to control, prevent, and eliminate it have been intensified in the past two decades which has contributed to reductions in malaria prevalence and under-five mortality. However, there was a 21% upsurge in malaria prevalence between 2010 and 2015. Zambia is one of the only 13 countries to record an increase in malaria among 91 countries monitored by the World Health Organization in 2015. This study investigated the upsurge by decomposition of drivers of malaria.

**Methods:**

The study used secondary data from three waves of nationally representative cross-sectional surveys on key malaria indicators conducted in 2010, 2012 and 2015. Using multivariable logistic regression, determinants of malaria prevalence were identified and then marginal effects of each determinant were derived. The marginal effects were then combined with changes in coverage rates of determinants between 2010 and 2015 to obtain the magnitude of how much each variable contributed to the change in the malaria prevalence.

**Results:**

The odds ratio of malaria for those who slept under an insecticide-treated net (ITN) was 0.90 (95% CI 0.77–0.97), indoor residual spraying (IRS) was 0.66 (95% CI 0.49–0.89), urban residence was 0.23 (95% CI 0.15–0.37), standard house was 0.40 (95% CI 0.35–0.71) and age group 12–59 Months against those below 12 months was 4.04 (95% CI 2.80–5.81). Decomposition of prevalence changes by determinants showed that IRS reduced malaria prevalence by − 0.3% and ITNs by − 0.2% however, these reductions were overridden by increases in prevalence due to increases in the proportion of more at-risk children aged 12–59 months by + 2.3% and rural residents by + 2.2%.

**Conclusion:**

The increases in interventions, such as ITNs and IRS, were shown to have contributed to malaria reduction in 2015; however, changes in demographics such as increases in the proportion of more at risk groups among under-five children and rural residents may have overridden the impact of these interventions and resulted in an overall increase. The upsurge in malaria in 2015 compared to 2010 may not have been due to weaknesses in programme interventions but due to increases in more at-risk children and rural residents compared to 2010. The apparent increase in rural residents in the sample population may not have been a true reflection of the population structure but due to oversampling in rural areas which was not fully adjusted for. The increase in malaria prevalence may therefore have been overestimated.

## Background

Malaria is endemic to Zambia and has stable transmission throughout the year [1], the burden of the disease follows a seasonal pattern that is dictated by environmental factors including rainfall, vegetation and temperature among others [2]. It is lowest during the cold and dry season around June to August and highest during the warm and rainy season from November to April [3].

Apart from following the seasonal pattern based on temperature and rainfall season, the malaria burden also follows regional trends. The northern parts of Zambia namely Luapula, Northern, North-western and Muchinga provinces receive the highest rainfall of up to 1200 mm annually and similarly have the highest malaria prevalence of above 20% in children aged below 5 years at the peak of the transmission season. The middle parts of Zambia from Eastern, Central, Copper-belt and Western provinces which receive between 800 and 1000 mm of rainfall annually also have low to moderate burden of malaria with prevalence from 10 to 20% whilst the southernmost parts of the country including Lusaka, Southern and the southern parts of Western provinces receive less than 800 mm of rainfall annually similarly have malaria prevalence rates of less than 10% malaria in under five children [4].

Interventions against malaria can alter the natural transmission cycle of malaria from the vector to the human host and vice versa and be able to change and/or eliminate malaria transmission altogether. A meta-analysis of 13 studies done in sub-Saharan Africa on indoor residual spraying (IRS) effectiveness indicated a relative risk in malaria prevalence of 0.38 [5]. A study in Zambia on the Copper-belt province reported an IRS protective incident rate ratio of 0.65 [6]. Use of insecticide-treated nets (ITNs) is another effective strategy for reducing malaria burden; one systematic review of over 10 studies in sub-Saharan Africa estimated an incident rate ratio of 0.49 [7]; in another systematic review which included 19 studies worldwide, ITNs were found to have a protective effect of 17% compared to no nets (Relative Rate 0.83) and to reduce the incidence of uncomplicated malaria episodes by 50% [8]. Another systematic review attributed 79% of the reduction in malaria burden to vector control interventions [9].

Prompt treatment of malaria cases with effective antimalarial medication and environmental management also reduces malaria morbidity and mortality; one study showed an odds ratio of 0.55 between 2003 and 2005 after the deployment of artemisinin-based combination therapy (ACT) and ITNs [10]. Another study, a systematic review in West Africa involving seven studies found that intermittent preventive therapy in children (IPT_c_) prevented three-quarters of all clinical malaria episodes [11]. At the time of the literature search, studies in Zambia which linked treatment to the burden of disease were not found.

Financial and programmatic coverage figures compiled by the World Health Organization (WHO) indicate that prevention and treatment interventions for malaria were implemented in Zambia at a larger scale during the period 2010 to 2015 than before [12]. Zambia implemented mass distribution of ITNs in 2012 and 2015 throughout the country [13] and routine ITN distribution to pregnant women and children below 5 years old, IRS, prompt diagnosis and treatment of malaria cases, intermittent prophylactic therapy for pregnant women and mass drug administration among other activities. Despite these interventions, the prevalence of malaria in Zambia increased during the same period from 16.0 to 19.4% [4], yet globally, the malaria incidence reduced on average by more than 21% between 2010 and 2015. Zambia is among the only 13 countries that recorded increased incidence out of the 91 countries and territories with malaria transmission in 2015 [12]. During the previous strategic planning cycle (2006–2010), malaria incidence in Zambia reduced by about 40.3% from 412/1000 persons to 246/1000 persons in 2009 [14]. This study investigated factors that could have contributed to malaria burden increase despite the high impact interventions implemented by government and cooperating partners in Zambia. The findings would inform malaria programme implementers, policymakers and donors alike on how to further strengthen the fight against malaria in Zambia and possibly plan to avoid future resurgences in malaria.

## Methods

### Study setting

Zambia is in Southern Africa and consists mostly of high plateaus with some hills and mountains dissected by river valleys. Its total landmass is 752,614 square kilometres; and has a tropical climate with rainfall ranging from 500 to 1400 mm annually and average temperatures are 20 °C; however, it has average temperatures above 30 °C for at least 8 months a year [15]. These conditions are favourable for endemic malaria transmission. Zambia’s mid-year population for 2015 was estimated at 15, 473,905 [16].

### Study design

This study is a secondary analysis of three Malaria Indicator Surveys (MIS) [4, 17, 18], which is a cross-sectional household survey.

### Sampling frame and sample size

Administratively the country is divided into ten provinces, and the provinces are further divided into districts; as of 2015, there were 104 districts in Zambia. For statistical purposes, each district is subdivided into census supervisory areas, and these are in turn subdivided into standard enumeration areas (SEAs); as of 2015, there were 25,631 SEAs in Zambia. Therefore, the sample frame of these surveys was the list of all SEAs developed from the preceding Population Census of 2010. From the selected SEAs, 25 households were selected without replacement. A two-stage sampling was used; the first stage was the selection of SEAs from the national listing of all SEAs by the Central Statistical Office by random computer selection and a second stage sampling in the field of 25 households from a listing of all households in each of the selected SEAs. Data was collected on all household members in the selected households, and blood samples for malaria testing were done for all children aged below 5 years.

The sample sizes used were determined using 95% confidence limits, 80% power, a design effect of 2.00, and 20% adjustment for non-response (from household refusals, or abandoned households). The population assumptions used were the prevalence of malaria and severe anaemia in under five children in previous MIS and an estimated 77% of households having at least a child aged less than 5 years old. For the 2015 MIS, the sample size was 3720 households and 150 SEAs, 2012 MIS 4000 SEAs and 160 SEAs while the 2010 MIS sampled 4500 households and 180 SEAs. This sampling procedure was in line with guidelines for MIS [19].

### Data collection

Datasets for the 2010, 2012 and 2015 waves of the MIS were obtained from the National Malaria Elimination Centre (NMEC). The primary data source surveys were collected from households between April and May in 2010 and 2012 and between April and June in 2015. Climatic data namely average temperature and cumulative rainfall in the 3 months prior to the studies were obtained from the satellite-generated climate database called Climate Explorer https://climexp.knmi.nl. The data was exported to Stata 15 for data cleaning and analysis.

### Data variables

The dependent variable was a binary variable equal to one if a child aged below 5 years had a positive malaria test and zero otherwise. Malaria parasite presence in capillary blood under microscopy in children aged below 5 years was decided by consensus among three experienced malaria slide readers at the central level. The predictor variables considered were age, gender, location of residence (urban vs rural), type of housing as a binary variable (standard house as one with solid roof of iron, asbestos or ceramic tiles, brick walls with closable windows and a concrete floor whilst houses not meeting this standard were considered non-standard). Also, wealth quintile on a 1 to 5 scale (1 being poorest and five being wealthiest), IRS sprayed *vs* non-sprayed households, ownership and coverage of all sleeping spaces in the household with bed-nets, and use of bed-net the previous night were included. The presence of fever among children in the 2 weeks preceding the survey, seeking treatment the same day or next day, testing by finger prick among those with fever in preceding 2 weeks to survey and treatment of malaria cases were also included. Rainfall, temperature and altitude were also included as they are documented key determinants of malaria. The predictor variables were selected based authors’ subject knowledge and literature review [20–22].

### Data analysis

Basic descriptive statistics of characteristics of respondents were described. Hypothesis tests such as Student’s T-tests for continuous variables; proportional tests, Chi square test for categorical variables to test the significance of the changes in variables over time and between groups of variables were done.

Further analysis using MIS primary datasets factored in the study design of two-stage sampling by use of weights and strata to account for differential population sizes and response rates in the clusters which were the primary sampling unit in the MIS [23]. Correlation among independent variables was done to identify covariates which were strongly correlated before fitting the model. Multivariable logistic regression was done to determine the effect sizes of the independent variables on the dependent variable, using primary MIS datasets for 2010, 2012 and 2015. To explain differential rises/drops in malaria prevalence at the provincial and national level over time, decomposition of the changes in malaria prevalence were done by calculating the marginal effects of each of the predictor variables using the margins command in Stata version 15. The marginal effect of an independent variable is the magnitude of the change in the dependent variable that a 1% change in the independent variable of interest would effect while holding other covariates constant. Using marginal effects provided additional information to the multivariable logistics model which gave odds ratios for malaria determinants, by providing contributions of each determinant to the changes in malaria prevalence across the successive surveys. The marginal effects of each variable were multiplied by the magnitude of the changes in coverage of the variable between 2010 and 2015 by subtracting its point estimate in 2015 from its point estimate in 2010 to estimate its contribution to the changes in malaria prevalence. STATA version 15 (Stata Corp, College Station, Texas, USA) software was used for analysis [24]. For all statistical tests, a P-value of < 0.05 was considered statistically significant.

## Results

### Respondents’ characteristics

Table 1 shows the basic characteristics of the respondents for the three MIS. The coverage and basic characteristics of the respondents for all three MIS are comparable across the years from 2010, 2012 and 2015 for gender, age groups and wealth index, however, there were more households and people in urban areas in 2010 (37% and 49%) compared to 2015 (23% and 22%), respectively.

**Table 1:** Summary of the respondents’ characteristics Source: MoH [4, 17, 18]

Characteristic	Category	MIS 2010	MIS 2012	MIS 2015
		Number (%)	Number (%)	Number (%)
Households	Urban	1595 (37)	750 (20)	815 (23)
	Rural	2766 (63)	3050 (80)	2728 (77)
Wealth index of households	Lowest	859 (20)	759 (20)	720 (20)
	Second	847 (19)	763 (20)	715 (20)
	Middle	868 (20)	758 (20)	716 (20)
	Fourth	907 (21)	758 (20)	709 (20)
	Highest	879 (20)	762 (20)	714 (20)
Individuals by residence location	Urban	9753 (49)	3425 (20)	3515 (22)
	Rural	10,289 (51)	13,503 (80)	12,614 (78)
Sex	Male	9753 (49)	8053 (48)	7520 (47)
	Female	10,289 (51)	8874 (52)	8606 (53)
Age group	Children (0–14 years)	9469 (48)	7922 (47)	7482 (46)
	Adults 15 and above years	10,582 (53)	9006 (53)	8644 (54)
Children	0–4 years	4008 (42)	3301 (42)	2822 (38)
	5–14 years	5452 (58)	4621 (58)	4660 (62)

### Changes in prevalence

There was a statistically significant increase in malaria prevalence between 2010 and 2015 (P value < 0.001), ITN utilization, IRS sprayed households, and temperature also increased significantly (P values < 0.001) while rainfall reduced significantly (P value < 0.001). Table 2 gives a summary of the changes in malaria prevalence and predictor variables between 2010 and 2015.

**Table 2:** Changes in key malaria variables between 2010 and 2015 Source MoH [4] and https://climexp.knmi.nl

Variable	2010	2015	P-value	Target
1. Malaria microscopy slide positive (%)	16.0	19.4	< 0.001	4.0
2. Age group				
0–11 months (%)	20.2	13.6	0.259	
12–59 months (%)	79.8	86.4		
3. Sex				
Male (%)	50.2	47.3	0.671	
Female (%)	49.8	52.7		
4. ocation of residence				
Rural (%)	70.9	80.9	0.098	
Urban (%)	29.1	19.1		
5. Type of house				
Makeshift house (%)	86.6	86.0	0.836	
Standard house (%)	13.4	14.0		
6. Altitude of house location (meters above sea level)	1153.0	1151.0	0.658	
7. Wealth status				
Poorest quintile (%)	26.9	20.6	0.321	
Not in poorest quintile (%)	73.1	79.4		
8. Slept under an ITN previous night (%)	49.9	58.9	< 0.001	80
9. Indoor residual sprayed household (IRS) (%)	23.1	28.9	< 0.001	100
10. Among under 5 children with fever in last 2 *weeks*				
Sought treatment within same or next day (%)	31.2	31.8	0.173	
Self-reported malaria tested (%)	16.7	35.5		100
Received antimalarial same or next day (%)	18.7	25.2		100
11. Temperature (°C)	20.5	21.5	< 0.001	
12. Rainfall (millimeters of water)	645.0	598.0	< 0.001	

**Fig. 1:**
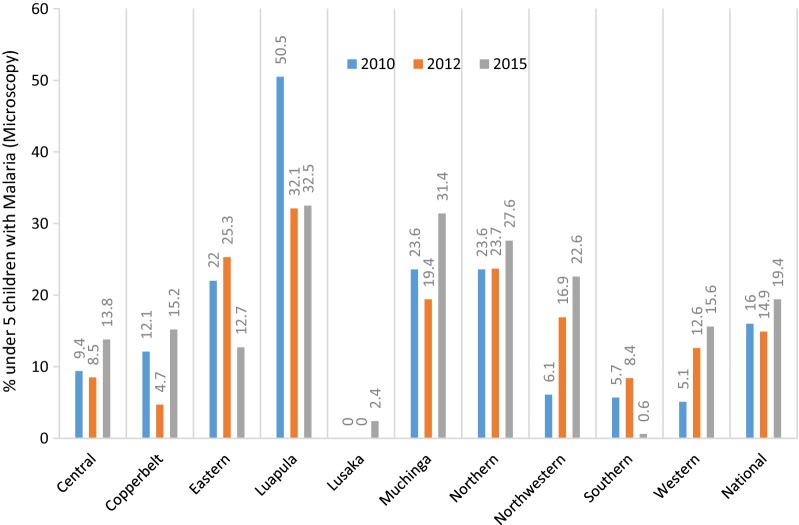
Malaria prevalence by province 2010–2015 [4]

Figure 1 summarizes the national and malaria provincial prevalence between 2010 and 2015. Seven (7) out of the ten (10) provinces (Central, Copper-belt, Lusaka, Muchinga, Northern, North-western and Western provinces) recorded increases in malaria prevalence while only three (3) recorded reductions (Eastern, Luapula and Southern provinces). The increases in Central, Copper-belt, Lusaka, Muchinga and Northern were not statistically significant while the increases in North-western and Western were statistically significant regarding the respective provinces’ 2010 baselines (P-value < 0.001).

### Determinants of malaria and decomposition analysis

Children aged 12 to 59 months were found to have adjusted odds ratio of 4.0 (95% CI 3.3–6.7) compared to their counterparts aged 0 to 11 months, urban location of residence had adjusted odds ratio of 0.23 (95% CI 0.14–0.37) compared to rural area, standard housing had adjusted odds ratio of 0.50 (95% CI 035–0.71), IRS 0.66 (95% CI 0.49–0.90) and sleeping under a net the previous night 0.90 (0.77–0.90). Table 3 shows unadjusted and adjusted odds ratios, P values and marginal effects of predictor variables. Increase in age was significantly associated with the increase in the risk of malaria positivity while increases in the percentage of children who reside in standard houses, urban location, IRS, and altitude were significantly associated with reduced risk of malaria positivity while ITN use, gender and temperature were not significantly associated with malaria positivity.

**Table 3:** Effect sizes and marginal effects of predictor variables on malaria Source: Authors’ secondary data analysis

Variable	Unadjusted		Adjusted		Marginal effects	
	Odds ratio (95% CIs)	P-value	Odds ratios (95% CIs)	P-values	Marginal effect (95% CIs)	P-value
Age (12–59 months)	4.15 (2.90–5.95)	< 0.001	4.04 (2.80 –5.81)	< 0.001	0.175 (0.128, 0.222)	< 0.001
Sex (female)	0.89 (0.76–1.05)	0.161	0.90 (0.75–1.08)	0.240	− 0.014 (− 0.036, 0.009)	0.245
Residence (urban)	0.18 (0.12–0.28)	< 0.001	0.23 (0.15–0.37)	< 0.001	− 0.182 (− 0.241, − 0.123)	< 0.001
Altitude	1.00 (0.99–1.00)	0.827	1.00 (0.99–1.00)	0.034	< − 0.001	0.032
Standard house (yes)	0.27 (0.20–0.37)	< 0.001	0.40 (0.35–0.71)	< 0.001	− 0.089 (− 0.132, − 0.043)	< 0.001
Wealth status (not poorest 5th)	0.44 (0.35 –0.55)	< 0.001	0.65 (0.52 –0.81)	< 0.001	− 0.055 (− 0.083, − 0.027)	< 0.001
Indoor residual spraying (yes)	0.61 (0.46–0.80)	0.001	0.66 (0.49–0.89)	0.008	− 0.051 (− 0.089, − 0.014)	0.007
Slept net previous night (yes)	0.78 (0.66–0.91)	0.002	0.90 (0.77–0.97)	0.192	− 0.013 (− 0.033, − 0.007)	0.193
Rainfall	1.00 (1.00–1.00)	< 0.001	1.00 (0.99–1.05)	0.250	< 0.001	0.250
Temperature	0.73 (0.66–0.80)	< 0.001	0.71 (0.62 –0.82)	< 0.001	− 0.042 (− 0.060,–0.025)	< 0.001

MIS year (2010 baseline)	Ref	Ref	Ref	Ref		
(2012)	0.82 (0.64–1.05)	0.122	1.11 (0.84–1.45)	0.460		
(2015)	1.48 (1.14–1.92)	0.003	1.69 (1.32–2.17)	< 0.001		

When the marginal effects of the predictor variables on malaria prevalence as estimated by the model were combined with actual coverage changes recorded in the MIS between 2010 and 2015, the changes in standard housing, IRS and urban residence, were found to be significant. The marginal effects of the significant predictor variables were standard house − 0.089 (95% CI − 0.141 to − 0.043), IRS − 0.051 95% (CI − 0.089 to − 0.014), urban residence − 0.182 (95% CI − 0.241 to − 0.123) and sleeping under a net at − 0.013 (95% CI − 0.033 to − 0.007). In other words, on a linearized scale and factoring in all other predictors included in the model and residuals, a 5.5% rise in urban residence or 11.2% rise in Standard Housing or 19.6% rise in Indoor Residual Spraying or an 80% increase in ITN use would result in a 1% decrease in malaria prevalence and vice versa. Table 4 shows how changes in predictor variables affected the changes in malaria prevalence by province and national level. Of note is that changes in IRS and ITNs each contributed − 0.3% and − 0.2% respectively to the changes in malaria prevalence between 2010 and 2015 while changes in rural location and age group 12–59 months contributed + 2.2% and + 2.3%, respectively.

**Table 4:** Observed changes versus estimated changes by predictor variables Source: Authors’ secondary data analysis

Province	Actual observed			Percentage point changes estimated by predictor variables									
	Baseline prev. 2010	2015 prev.	Absolute % change	IRS	Standard housing	Residence	Wealth status	Age group 12 –59 mo.	Sleptnet	Altitude	Sex	Temp.	Total
Central	9.4	13.8	4.4	− 0.5278	− 0.174	− 0.394	0.152	2.460	0.0575	− 0.002	− 0.04	− 0.07	1.455
Copper-belt	12.1	15.2	3.1	− 0.0058	− 0.696	6.501	0.115	2.362	− 0.1175	0.001	− 0.02	− 0.06	8.086
Eastern	20.3	12.7	− 7.6	− 2.4534	− 0.174	− 1.182	0.471	1.673	− 0.3275	0.001	0.045	− 0.10	− 2.050
Luapula	50.5	32.5	− 18	− 0.8004	− 0.174	− 1.379	− 0.925	2.558	− 0.935	− 0.004	− 0.04	− 0.03	− 1.724
Lusaka	0.0	2.4	2.4	0.841	1.566	7.486	− 0.060	2.755	− 0.1125	0.010	− 0.09	− 0.10	12.298
Muchinga	21.8	31.4	9.6	− 0.8352	− 0.348	1.97	− 0.864	1.968	− 0.3875	− 0.011	0.05	− 0.03	1.509
Northern	26.1	27.6	1.5	− 0.2436	− 1.131	− 2.758	− 0.914	2.362	0.0425	− 0.002	− 0.11	− 0.03	− 2.784
Northwestern	5.1	22.6	17.5	− 0.9802	1.044	0.788	− 1.031	2.165	0.255	− 0.004	0.08	− 0.01	2.300
Southern	5.7	0.6	− 5.1	− 0.1276	− 0.261	1.379	− 0.733	2.165	− 0.235	− 0.001	− 0.11	0.04	2.118
Western	5.1	15.6	10.5	0.5742	− 0.087	0.394	− 1.007	2.558	0.32	0.002	− 0.06	− 0.07	2.632
National	16	19.4	3.4	− 0.336	− 0.026	2.167	− 0.340	2.325	− 0.195	− 0.001	− 0.04	0.043	3.593

## Discussion

Changes in malaria prevalence by province showed that seven out of ten provinces in Zambia recorded increases in malaria prevalence in 2015 compared to the baseline of 2010; however, this does not explain why there were increases where there were increases. Our findings suggest that significant predictors of malaria prevalence include age, the location of residence, altitude, housing, IRS, and temperature. Increase in age was significantly associated with the increase in the risk of malaria prevalence while increases in the percentage of children who reside in standard houses, urban location, IRS, and altitude were significantly associated with reduced risk of malaria prevalence while gender, ITN use and rainfall were not significantly associated with malaria prevalence.

There were conflicting results among studies conducted in Zambia on the determinants of malaria prevalence; some studies found ITNs and IRS as effective [25, 26], but another study found that both IRS and ITNs were not significant predictors of malaria prevalence among children [27]. This study may have detected significance because MIS was highly powered and nationally representative compared to other studies. The study also agreed with similar studies in Sub-Saharan Africa using MIS datasets which found that IRS was effective [28, 29], in meta-analyses [30] and those targeting specific interventions, such as IRS [5], and climatic factors such as rainfall and temperature [2, 31].

Whilst this study found temperature to be a significant predictor but not rainfall, it is probably because only 3 months of rainfall prior to the surveys were considered in the model so this is likely not to have factored in seasonal variability of malaria where rainfall is an established significant predictor of malaria [31–33].

Decomposition of malaria prevalence by determinants showed that the increases in the two mainstream malaria interventions ITN and IRS between 2010 and 2015 could have possibly contributed to the reduction in the overall malaria prevalence by − 0.2% and − 0.3%, respectively, however, these positive contributions were overridden by changes in other factors which contributed to the increase namely location of residence in rural areas and changed composition of under-five children by age groups which contributed + 2.2% and + 2.3%, respectively.

Residing in urban areas carried less risk of malaria prevalence compared to rural areas with adjusted odds ratio of 0.23 (0.14–0.37) implying that the 10% replacement of less at risk urban residents in 2010 with 10% more at risk rural residents in 2015 contributed to the overall increase in malaria prevalence in 2015, similarly there were 6.6% more children in the age group 12–59 months with an adjusted odds ratio of 4.0 compared to the less-at-risk children in the age group 0–11 months. Other than poor housing structures and wealth status in rural areas compared to urban areas which were adjusted for in this study, other factors still make rural residents more at risk of malaria than their urban counterparts such as outdoor activities for example farming, fishing, animal rearing which have been shown in literature to increase vector-human contact [34]. Under five children are generally more at risk of malaria than the general population [35], however, infants (age group 0–11 months) were less at risk of malaria positivity in this study compared to older under five children aged 12–59 months, this was similar to what was found in Uganda [28] and the Democratic Republic of Congo [36]. It is documented that babies acquire passive antibodies from their mothers in utero and during breastfeeding against malaria which gives some protection in early infancy; however, these wane off around 6 months of age [37]. Secondly, as the children grow older, they become more independent of their caregivers and may move outdoors more frequently and wear less protective clothing against invading mosquito compared to infants [38].

This study was of a cross-sectional design in nature and, therefore, was not meant to explain population dynamics such as changes in urban–rural migration or age differences among under-five children, however it was noted that the sampling of clusters in 2015 MIS had more clusters in rural areas (77%) compared to the cluster sampling in 2010 which had 58% of clusters in rural areas. The 2015 MIS cluster sampling was based on the 2010 Census of Population and Housing population structure while the 2010 MIS cluster sampling was based on the 2000 Census of Population and Housing structure. Whilst urbanization was projected to increase in Zambia from 39.5 in 2010 to 41.8% in 2015 [16], the fertility rates in rural areas (6.6%) were higher than in urban areas (3.7%) [39] so this could also explain why there were more children in rural areas than urban areas. Further, birth rates were reducing over the years in Zambia, for example, the crude birth rate was 44.3/1000 in 2011, but decreased to 42.4/1000 in 2015 [16]; this could explain why there was a lower proportion of under-five children aged below 1 year in 2015 compared to 2010.

The ‘upsurge’ in malaria prevalence noted in the 2015 MIS compared to 2010 MIS may not truly represent a rise in prevalence in Zambia; the true 2015 MIS prevalence may have been over-estimated because of over-sampling the rural areas where there was a higher burden of malaria. Whilst sampling weights were applied in both 2010 and 2015 because of oversampling in rural areas, 2010 reverted the oversampling to true rural population proportion from 70.8 to 68.3% which was not statistically different from 65.3% reported in the 2000 census; however, 2015 did not adequately revert the rural strata proportion to the truly representative rural proportion. Before weights were applied, the rural percentage among under-five children was 81.2%, after weights were applied, the rural stratum percentage reduced to 79.0% which was statistically different from the 60.5% reported in the 2010 census of population and housing. In this case, there would have needed to standardize the findings to the 2010 population structure, and if this were done, the malaria prevalence for 2015 would have been about 17.6%. The 17.6% is not statistically different from the 16.0%-point prevalence reported in the 2010 MIS (P-value = 0.398). Though there were changes in climatic indicators such as rainfall and temperature, this study did not elicit sufficient evidence based on their marginal effects to suggest that they influenced the upsurge in malaria between 2010 and 2015. Rainfall, temperature and altitude are well documented factors that influence malaria [31, 33] as they are known to affect vector dynamics, such as vector population density and biting behaviour driven by reproduction needs [32, 40] and also influence human behaviour that predisposes them to bites by the vector, for example, when it is hot, some people in villages sleep outside and avoid to cover themselves with mosquito nets because of the heat [41, 42].

## Conclusion

The determinants that were found to be significantly associated with malaria include age, the location of residence, altitude, housing, IRS and temperature. Malaria prevalence recorded in Zambia increased in 2015 by 21% of the 2010 baseline despite overall increases in mainstream interventions such as IRS and ITN utilization partly due to increases in a high-risk group of children aged between 12 and 59 months and increases in more at risk rural residents. The findings of this study demonstrate the complexity of the drivers of malaria; while interventions like ITNs and IRS have been demonstrated to bring down malaria, counter changes in demographics such as increases in more at risk groups among under-five children and increases in the ratio of rural–urban residence may override the impact of these interventions. The apparent increase in rural residents in the 2015 sample population may not have been a reflection of the true population proportion of rural areas but due to oversampling of the rural strata that was not adequately adjusted for using weights. The true malaria point prevalence may, therefore, have been overestimated.

Though there was an overall ITN utilization increase of 10% between 2010 and 2015 among under-five children, the overall ITN utilization rate of 58.9% was still below the national target of 80%. Similarly, the overall coverage of IRS was low at below 30% at national level despite some provinces having coverage as high as 60%; however, this is below desired coverage of 80% or more.

Standard housing which consists of a solid roof, brick walls with closable windows and concrete floor has been shown to be an effective way of addressing malaria after adjusting for all other factors like wealth status, urban/rural residence, IRS, ITN utilization among others.

### Limitations

Provincial data on prompt treatment within the same day/next day was insufficient in most provinces thus the effect of prompt treatment on malaria prevalence was not included in our multivariable logistic model. In other areas such as Myanmar, early diagnosis and treatment were found to be more effective in preventing malaria than the deployment of ITNs in a randomized cluster trial as vectors bite early in the evening before ITNs can protect residents [43].

This study, therefore, missed an opportunity to evaluate a key element of the malaria control/elimination strategy due to insufficient data at the provincial level from the Malaria Indicator Surveys.

It is important to note that this study could have underestimated the effect sizes of ITN, IRS and temperature as it only used sleeping under an ITN the previous night which does not include the mass effect of ITNs in areas where there was high coverage. Similarly, sleeping in a household that was indoor residual sprayed does not include the mass effect of high IRS coverage in an area. Further, minimum temperature has been shown to an effect on malaria while in our study we used average monthly temperatures 3 months prior to the survey. Despite these limitations, this study still found that the size effects for IRS and temperature were significant.

The baseline data for Muchinga province used data from the Northern Province and as such may not have been too accurate in the secondary data analysis as Muchinga province was not detached from Northern Province at the time. Interpretation of any increases from Northern and Muchinga provinces, therefore, need to be handled with caution.

## Abbreviations

ACT: artemisinin-based combination therapy
IPT: intermittent presumptive therapy
IRS: indoor residual spraying
ITN: insecticide-treated net
LLIN: long-lasting insecticide-treated net
MIS: Malaria Indicator Survey
MoH: Ministry of Health
NMCP: National Malaria Control Programme
NMSP: National Malaria Strategic Plan
RBM: roll back malaria
RDT: rapid diagnostic tests
SEA: standard enumeration area
UNDP: United Nations Development Programme
WHO: World Health Organization
